# rs61991156 in miR-379 is associated with low capability of glycolysis of gastric cancer by enhanced regulation of PKM2

**DOI:** 10.1186/s12935-018-0593-0

**Published:** 2018-07-04

**Authors:** Na Cao, Meng Li, Jun Han, Yongren Wang, Xiaowei Wang

**Affiliations:** 1 Department of Medical Affairs, Nanjing Center Hospital, Nanjing, China; 2 Department of Clinical Laboratory, Children’s Hospital of Nanjing Medical University, Nanjing, 210008 China; 3 Department of Hematology and Oncology, Children’s Hospital of Nanjing Medical University, Nanjing, China

**Keywords:** Gastric cancer, PKM2, Glycolysis, SNP, miRNA

## Abstract

**Background:**

Glycolysis is an important metabolic oncogenic change also play a pivot role in the Warburg effect. Glycolysis related gene PKM2 expressed differently individually. Presently, we sought to investigate the effect of single nucleotide polymorphism (SNP) at rs61991156 of miR-379 on gastric cancer (GC) proliferation and metabolism.

**Methods:**

The genotype of rs61991156 in miR-379 was investigated by using real-time PCR. The glycolysis-related metabolites were determined by using GC–TOF–MS. The biological effects of rs61991156 in miR-379 was explored by in vitro studies.

**Results:**

In this study, we found that rs61991156 in miR-379 was involved in the occurrence of GC by acting on the 3′UTR region of PKM2. The clinical data analysis revealed that A > G in rs187960998 was significantly associated with better differentiation, small tumor size, and non-metastasis. In vitro study further revealed that A > G SNP of miR-379 could decrease GC cell proliferation as well as the promoter activity and expression of PKM2. The glycolysis of the patients with miR-379 GG genotype was significantly lower than AG and AA genotype by metabolomics analysis. The patients with AA genotype have significantly lower PKM2 expression compared to the G carrier, while there is no significant expression difference in miR-379 expression. Patients with AA genotype have significantly shorter survival rate compared to the G carrier.

**Conclusion:**

rs61991156 in miR-379 was highly associated with a decreased risk, well differentiation and better post-surgery survival in Chinese population by inhibiting the expression of PKM2.

**Electronic supplementary material:**

The online version of this article (10.1186/s12935-018-0593-0) contains supplementary material, which is available to authorized users.

## Background

The Warburg effect is observed in most cancer cells, reflected as predominantly producing energy at a high rate of glycolysis followed by lactic acid fermentation. However, most normal cells demonstrated a comparatively low rate of glycolysis followed by oxidation of pyruvate in mitochondria. Malignant, rapidly growing tumor cells typically have glycolytic rates up to 200 times higher than those of their normal tissues of origin; this occurs even if oxygen is plentiful [1–3]. In the other point of view, the glycolysis is positively correlated to the tumor growth and malignancy. Today, mutations in oncogenes and tumor suppressor genes are thought to be responsible for malignant transformation, and the Warburg effect is considered to be a result of these mutations rather than a cause [2].

Since the glycolysis provides most of the building blocks required for cell proliferation, cancer cells (and normal proliferating cells) have been proposed to need to activate glycolysis, despite the presence of oxygen, to proliferate [4]. A set of genes regulated the process of glycolysis, three of them are most important. They are hexokinase (HK), phosphofructokinase-1 (PFK1) and pyruvate kinase (PK), in which PKM2 is the last step within glycolysis and was detected to expressing in most cancers [5].

Similar to other genes, PKM2 was regulated by miRNA. It has been reported that PKM2 can be targeted by the tumor-suppressive miRNA including miR-326 [6], miR-122 [7, 8], miR-124 [9], miR-137 [9], etc., thus to decrease the Warburg effects. However, although the expression of these miRNAs was detectable within tumors, over-expression of their targeting gene: PKM2 still appeared frequently. There are many explanations, in which SNP in miRNA, especially mature miRNA are one of the convincing reasons.

MicroRNAs (miRNAs) are endogenous 22 nt non-coding RNAs which play important regulatory roles in animals and plants by targeting 3′UTR of mRNAs for cleavage or translational repression [10, 11]. Some SNPs in pre-microRNAs, flanking regions or target sites have been demonstrated to affect certain physiological processes or related to diseases [12]. In the present study, we found one possible valuable SNP in the mature miR-379, in which could potentially affect the binding ability to PKM2. We postulated that this SNP might contribute to the various expression of PKM2 within invidious and further affects the metabolism and growth of the tumor.

## Materials and methods

### Clinical sample information

The hospital-based case–control study consists of 871 GC patients and 812 cancer-free controls. All the subjects were recruited from the Center Hospital of Nanjing between January 2012 and January 2016. Patients with other hematological disorders, previous history of cancers, and chemotherapy were excluded. This study was approved by the Ethics Review Board of Hospital of Nanjing, and every patient had written informed consent. The clinical features of all the cases and controls were presented in Table 1.

**Table 1:** Clinical characteristic of gastric cancer patients and cancer-free controls

Features	Cases (*n* = 871)		Controls (*n* = 812)		*P*
	*N*	%	*N*	%	
Age (years)					0.805
≤ 50	376	43.17	345	42.49	
> 50	495	56.83	467	57.51	
Gender					0.248
Male	318	36.51	319	39.29	
Female	553	63.49	493	60.71	
*H. pylori* infection					< 0.0001
Positive	664	76.23	189	23.28	
Negative	207	23.77	623	76.72	
Differentiation					
G1	129	14.81			
G2	251	28.82			
G3	245	28.13			
G4	219	25.14			
Gx	27	3.10			
Tumor size (cm)					
≤ 5	587	67.39			
> 5	284	32.61			
Metastasis					
Yes	412	47.30			
No	459	52.70			

### Cell lines and cell culture

Gastric cancer cell lines including MKN-45 and AGS were purchased from American Type Culture Collection (ATCC). All cells were cultured in Dulbecco modified Eagle medium (DMEM) purchased from Gibco (CA, USA) supplemented with 10% fetal bovine serum (Invitrogen, Carlsbad, USA) and maintained in humidified 5% CO_2_ at 37 °C.

### Construction of luciferase-based reporter plasmids

The full length of PKM2 cDNA as well as its 3′UTR were synthesized and sub-cloned into pGL3 plasmids. For the gene promoter activity assays, 3′UTR of PKM2 was synthesized and sub-cloned into a pGL3 plasmid (Promega, WI, USA). The construction containing different genotype of miR-379 was also synthesized and cloned into pSilence 2.1-U6. All the DNA synthesis and clones were performed in Genscript Co. (Nanjing, China).

### Dual-luciferase reporter assay

The treated cells harvested 48 h after miRNA treatment, and the firefly luciferase expression was measured and normalized to Renilla activities. Dual-luciferase assays (Promega, Madison, WI) were performed according to the manufacturer’s protocol and detected with a Fluoroskan microplate reader (Thermo Labsystems, Helsinki, Finland). Transfection was repeated three times in triplicate (Additional file 1).

### Cell proliferation assays

Cell proliferation was determined by using CCK-8 (Dojin Laboratories, Kumamoto, Japan) according to the manufacturer’s instructions. Briefly, the control and infected cells were seeded at a density of 1 × 10^3^ cells/well in 96-well plates. 10 μL of CCK-8 was added to each well containing 100 µL of the culture medium, and the plate was incubated for 2 h at 37 °C. The viability of cells was evaluated by measuring the absorbance at 450 nm, using a microplate reader (Thermo Labsystems, CA).

### Genotype

Genomic DNA was extracted from peripheral blood by using QIAamp DNA blood mini kits (Qiagen, Hilden, Germany) according to the manufacturer’s instructions. Genotyping was performed with the TaqMan SNP Genotyping Assay. The PCR reactions were carried out in a total volume of 5 μL containing TaqMan Universal Master Mix, SNP Genotyping AssayMix, DNase-free water and genomic DNA. The PCR conditions were 2 min at 50 °C, 10 min at 95 °C, followed by 40 cycles at 95 °C for 15 s and 60 °C for 1 min. The 384-well ABI 7900HT real-time PCR system was applied (ABI, CA, USA).

### Immunohistochemistry (IHC)

Sections were stained according to the previous publication [7]. The section was incubated within primary mouse anti-human Ab for PKM2 (ab38237), the sections were stained with DAB according to manufacturer’s protocols and mounted and photographed using a digitalized microscope camera (Nikon, Tokyo, Japan).

### Plasma samples preparation and analysis by GC–TOF–MS

Metabolites extracted from plasma samples were analyzed using an Agilent 7890N gas chromatograph coupled with a Pegasus HT TOF mass spectrometer (Leco Corporation). Briefly, a 1 μL aliquot of the derivatized solution was injected with the splitless mode. Rxi-5 ms capillary column (30 m × 250 μm I.D., 0.25-μm film thickness; Restek Corporation, Bellefonte, PA, USA) was used for metabolites separation, with helium as the carrier gas at a constant flow rate of 1.0 mL/min. The temperature settings for injection, transfer interface, and ion source were 260, 260, and 210 °C, respectively. The separation was achieved with the following GC temperature program: 80 °C for 2 min, 10 °C/min to 220 °C, 5 °C/min to 240 °C, and 25 °C/min to 290 °C, and kept at 290 °C for 8 min. The data was collected with full scan mode (m/z 40–600), and an acquisition rate of 20 spectra/s. Electron impact ionization (70 eV) was used.

### Metabolomics data analysis

The data from GC–TOFMS was processed with ChromaTOF software (v4.22, Leco Co., CA, USA). Compound annotation was performed by comparing the mass fragments with NIST 08 Standard mass spectral databases with a similarity of more than 70% and finally verified by available reference standards. The annotated compounds from GC–TOFMS were imported to SIMCA-P software 12.0.1 (Umetrics, Umeå, Sweden) for multivariate statistical analysis. Supervised orthogonal partial least squares-discriminant analysis (OPLS-DA) was used to compare between groups. Differential metabolites were selected based on the criteria of variable importance in the projection (VIP) > 1 in OPLS-DA model and P value < 0.05 from Student’s t test.

### Statistical analysis

All experiments were performed in triplicate and repeated at least three times. Data were expressed as mean ± SD. The association between rs61991156 genotypes and the risk of GC was evaluated by calculating the odds ratios (ORs) and their 95% confidence intervals (CIs) using univariate and multivariate logistic regression analysis. Differences between two independent groups were tested with Student’s t test. All statistical analyses were carried out using SPSS version 18.0 and presented with Graph-pad prism software. Kaplan–Meier survival curves were plotted, and the log-rank test was done. The significance of various variables for survival was analyzed by the Cox proportional hazards model in a multivariate analysis. The results were considered to be statistically significant at P < 0.05.

## Results

### Clinical significance of rs61991156 in human gastric cancer

Total 871 GC cases and 812 healthy controls were involved in our study, and their clinical characteristics were listed in Table 1. There is no significant difference in “age” and “Gender” between the case and control, significantly more *H. pylori*-infected cases were involved compared to the controls. The GC patients were divided into four groups according to the pathological differentiation degree staged from G1 to G4.

As listed in Table 2, Chi square statistical analysis results showed that the genotypes of *rs61991156* were in Hardy–Weinberg equilibrium distribution pattern in the healthy control group (P = 0.52). Further, logistic regression analysis results revealed that the AG genotype and GG genotype presented a significantly decreased risk compared with AA genotype (AA vs. AG: odd ratio (OR) = 2.64; 95% CI 1.02–1.34; AA vs. GG: OR = 0.60, 95% CI 1.12–1.31; AA vs. G carrier: OR = 3.04, 95% CI 1.19–1.29). In addition, we performed Fisher exact the G carrier also indicated a decreased risk (P < 0.0001). All ORs were adjusted for sex, age, drinking history or family cancer history.

**Table 2:** Genotype frequencies of the miR-379 at rs61991156 polymorphism among GC cases and controls

Genotype	Cases (*n *= 871)		Controls (*n *= 821)		OR (95% CI)^a^	*P* value^a^
	*N*	%	*N*	%		
rs61991156						
AA	278	31.92	312	38.42	1	< 0.0001
AG	420	48.22	178	21.92	2.64 (1.02–1.34)	
GG	173	19.86	322	39.66	0.60 (1.12–1.31)	
G carrier	593	68.08	219	26.97	3.04 (1.19–1.29)	< 0.0001

^a^ The ORs, 95% CIs and *P* value were calculated after adjusting for age, gender, parental *H. Pylori* infection history and family cancer history

### Stratified analysis of the correlation between miR-379 SNP and GC

Next, we conducted the stratified analysis to investigate the correlation between the SNP *rs61991156* of miR-379 with the different clinical characteristics of GC which listed in Table 3. We found significant associations between rs61991156 genotypes with *H. pylori* infection, the tumor size, differentiation degree, and metastasis.

**Table 3:** Stratified analysis of SNP rs61991156 with clinicopathological parameters of GC

Features	Genotype				
	AA	AG	GG	AG vs. GG *P* value*	AA vs. GG *P* value*
Age (years)				0.2366	0.4974
≤ 50	121	174	81		
> 50	157	246	92		
Gender				0.7033	0.0931
Male	119	139	60		
Female	159	281	113		
*H. Pylori* infection				0.0728	< 0.0001
Positive	172	341	151		
Negative	106	79	22		
Differentiation				< 0.0001	< 0.0001
G1	6	64	59		
G2	61	125	65		
G3	96	139	10		
G4	94	89	36		
Gx	21	3	3		
Tumor size (cm)				0.587	< 0.0001
≤ 5	102	341	144		
> 5	176	79	29		
Metastasis				< 0.0001	< 0.0001
Yes	164	202	46		
No	114	218	127		

* Two-sided Chi square test for either genotype distributions or allele frequencies between cases and controls

### The role of rs61991156 in regulatory effects of miR-379 on PKM2 expression and cell proliferation in vitro

**Fig. 1:**
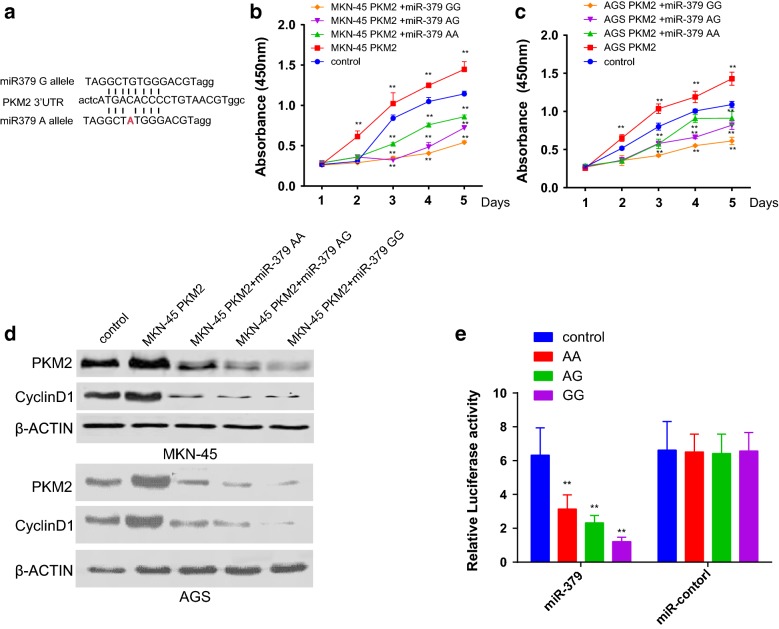
The role of rs61991156 in regulatory effects of miR-379 on PKM2 expression and cell proliferation in vitro. **a** Schematic diagram of rs61991156 SNP in miR-379 targeting PKM2 3′UTR. **b**, **c** Comparison of cell proliferation of MKN-45 and AGS human GC cell lines treated differently indicated in the figure. **d** MKN-45 and AGS were treated as it indicated in the figure, the expression of PKM2 and CyclinD1 were detected by western-blot. **e** Gene reporter assay was performed in MKN-45 cell, pGL-3 vectors containing 3′UTR of different genotype were co-transfected with both miR-379 and control. Data were presented as the mean ± SEM. *Indicated *P *< 0.05 and **indicated *P *< 0.01

Since the SNP rs61991156 was predicted to be located in the binding site of miR-379 on 3′UTR of PKM2 (Fig. 1a), also, previous studies revealed that PKM2 had proliferation promotion effects [13], we proposed that SNP of rs61991156 might affect the GC cell proliferation through the regulation of PKM2. We thus first constructed GC cell lines capable of overexpression of PKM2 regulated by its 3′UTR. Next, we detected the cell proliferation of all cell lines with the transfection of miR-379 with different genotypes. It turned out that the cell proliferation affected by PKM2 can be attenuated by miR-379, and the suppression effects were different within three genotypes. Suppression effect was weakest in AA genotype and was strongest in GG group, which reflected in PKM2 and cyclinD1 expression detected by western-blot (Fig. 1b–d, Additional file 1: Table S1). Next, we cotransfected the PGL-3 plasmid containing the 3′UTR of PKM2 as well as different genotypes of miR-379 into the GC cell lines. We found the PKM2 promoter activity can be regulated by its 3′UTR and miR-379, among which GG genotype decreased the promoter activity most significantly while AA genotype was the weakest. And mutation of miR-379 potential binding site of PKM2 3′UTR result in no apparent promoter activity decreases with the of miR-379 treatment with different genotypes (Fig. 1e, Additional file 1: Table S2).

### The role of rs61991156 in regulatory glycolysis and other related metabolomics in GC patients

**Fig. 2:**
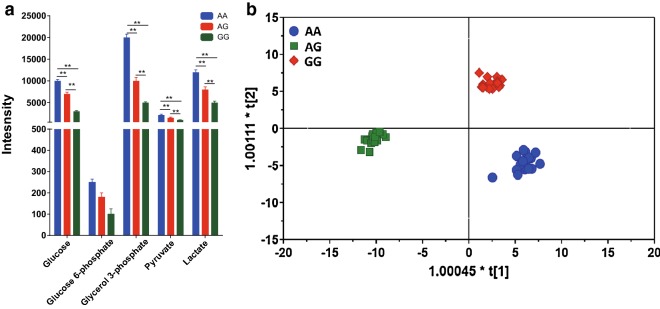
The role of rs61991156 in regulatory glycolysis and other related metabolomics in GC patients. **a** Comparison of glycolysis-related metabolites in different genotypes. **b** OPLS-DA scores plot showed a distinct plasma metabolic profile between among AA, AG and GG (R2X = 0.561, R2Y = 0.978, Q2(cum) = 0.939). Data were presented as the mean ± SEM. *Indicated *P *< 0.05 and **indicated *P *< 0.01

Orthogonal partial least squares-discriminant analysis (OPLS-DA) was performed to see whether there are metabolic differences among AA, AG and GG groups (Fig. 2a, Additional file 1: Table S3). As a result, OPLS-DA scores plot established with the identified endogenous plasma data (R2X = 0.561, R2Y = 0.978, Q2(cum) = 0.939) showed a clear class separation among the three groups (Fig. 2b). The metabolites involved in glycolysis were significantly altered among three groups with AA has the highest level and GG the lowest.

### A/G SNP was associated with low expression of PKM2 and longer postoperative survival in gastric cancer

**Fig. 3:**
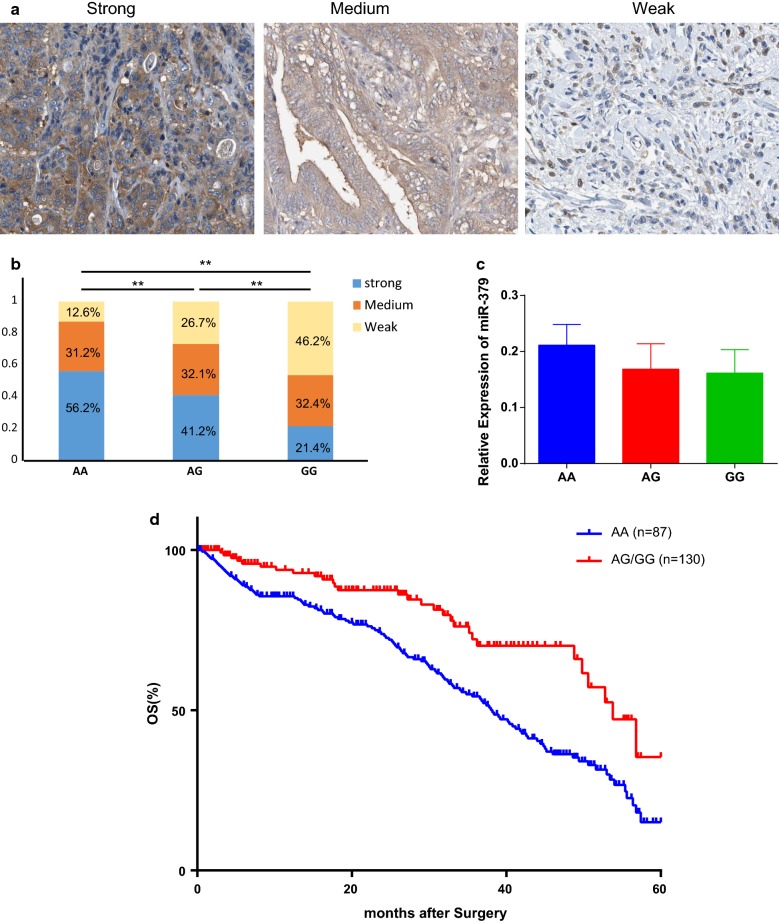
A/G SNP was associated with low expression of PKM2 and longer post-operative survival in gastric cancer. **a** Representative figures for IHC staining of PKM2 in GC tumor slide section. **b** Comparison of components of IHC staining in both in AA AG and GG genotype GC patients. **c** The expression level of PKM2 and miR-379 were determined by real-time PCR in AA AG and GG genotype GC patients. **d** Overall survival rate (OS) of post-surgery GC patients were analyzed by Kaplan–Meier survival curves between AA and G carrier. Data were presented as the mean ± SEM. *Indicated *P *< 0.05 and **indicated *P *< 0.01

We also confirmed the expression of PKM2 in clinical samples with different genotypes of rs61991156. PKM2 expression was detected in human gastric cancer by IHC. Within total 871 GC patients, we selected 100 cases for IHC staining of PKM2. The IHC staining consistency in miR-379 AA group was significantly different to AG and GG group (strong 56.2%, medium 31.2% and weak 12.6% for AA group; strong 41.2%, medium 32.1% and weak 26.7% for AG group; and strong 21.4%, medium 32.4% and weak 46.2% for GG group P < 0.001) (Fig. 3a, b). We then used real-time PCR and further confirmed the difference in PKM2 transcription between AA, AG and GG groups, there is no significant difference in miR-379 expression between these groups (Fig. 3c). Among of total 871 GC patients, we have 217 patients with follow-up data of survival, and these persons can be further divided in AA (n = 87) and GA/GG (n = 130). The 5-year survival rate in the AA group was only 4.35%, which was significantly lower than in the GA/GG group with a survival rate of 38.6% (HR = 2.019, P = 0.004) (Fig. 3d, Additional file 1: Table S4).

## Discussion

The general point of views on miR-379 were controversial. Some reports regarded it as a tumor suppressor which capable of down-regulating many oncogenes by targeting their 3′UTR region. For example, miR-379 was reported to be boosted by rifampicin and blocking the expression of ABCC2 (multidrug resistance-associated protein 2) thus to sensitize the HCC cells to chemotherapy [14]. Others also indicated that miR-379 was overexpressed in malignancies, and might serve as an indicator for bad prognosis. miR-379 expression was elevated in bone-metastatic prostate cancer cell lines and tissues. The expression of miR-379 was also correlated with shortened progression-free survival of patients with prostate cancer [15]. In our study, the results revealed that miR-379 was a tumor suppressor in human GC. From the clinical investigation, miR-379 GG genotype was associated with small tumor size, well differentiation, and non-metastasis which is related relatively low expression of PKM2 in gastric cancer. In line with the expression level, the glycolysis level within GC patients with GG genotype was also the weakest. We speculated that these results might result from rs61991156 in the mature form of miR-379.

In the present study, we found the SNP rs61991156 located within the mature form, generating three different genotypes of miR-379, among which A > G mutation generate an 8-mer complementary sequence in the 3′UTR of PKM2. The G allele might have significantly stronger the binding affinity to 3′UTR of PKM2 compared to the A allele. We thought this was the reason why miR-379 GG can significantly decrease the expression of PKM2 and in turn to attenuate both the proliferation and glycolysis of GC cells. Similar results concerning miRNA SNP have been reported previously resulting in either “Gain” or “LOSS” regulation of the targeting genes. miR-SNPs in miR-125a and Kaposi’s sarcoma-associated herpes virus-encoded miR-K5 were reported to impair miRNA processing by the Drosha/DGCR8 complex [16, 17]. SNP of miR-196a2 at rs11614913 in the mature miR-196a2 was reported to be associated with a significantly decreased rate of survival in individuals with non-small cell lung cancer, and the same research team of this study also suggested an association of rs11614913 with enhanced processing of mature miR-196a [18]. miR-146a-SNP (rs2910164) within the pre-miR-146a sequence reduced both the amount of pre- and mature miR-146a and apparently affected the Drosha/DGCR8 processing step [18, 19]. miR-196a2-SNP, miR-146a-SNP, miR-149-SNP (rs2292832), and miR-499-SNP (rs3746444) are each associated with increased breast cancer risk [20].

## Conclusion

So far, there is almost no report concerning the SNP of miR-379, we reported firstly that A > G SNP in 12nt of miR-379 might enhance the binding affinity to the 3′UTR of PKM2, thus to might be associated with low glycolysis level, well differentiation, as well as slower tumor growth. And the detection of rs61991156 might be associated with low occurrence and less aggressiveness of gastric cancer in Chinese population due to the enhanced regulations on PKM2.

## Additional file

**Additional file 1: Table S1.** Raw data for proliferation determination for MKN-45 and AGS with different treatment. **Table S2.** Raw data for luciferase assay. **Table S3.** Raw data for metabolites determination for various genotypes. **Table S4.** Survival analysis for the post-surgery patients with different genotypes.
